# Ideational factors and their association with insecticide treated net use in Magoe District, Mozambique

**DOI:** 10.1186/s12936-022-04405-1

**Published:** 2022-12-17

**Authors:** Joshua O. Yukich, Paul Hutchinson, Baltazar Candrinho, Jessica Butts, Filipe Murimirgua, Thomas P. Eisele, Rose Zulliger

**Affiliations:** 1 Center for Applied Malaria Research and Evaluation, Department of Tropical Medicine, Tulane University School of Public Health and Tropical Medicine, New Orleans, LA USA; 2 Center for Applied Malaria Research and Evaluation, Department of Global Community Health and Behavioral Science, Tulane University School of Public Health and Tropical Medicine, New Orleans, LA USA; 3 National Malaria Control Programme, Ministry of Health, Maputo, Mozambique; 4 U.S. President’s Malaria Initiative, Malaria Branch, US Centers for Disease Control and Prevention, Atlanta, GA USA; 5 Malaria Programme, Provincial Department of Health, Tete, Mozambique; 6 U.S. President’s Malaria Initiative, Malaria Branch, US Centers for Disease Control and Prevention, Maputo, Mozambique

**Keywords:** Malaria, Insecticide treated bed-nets, Ideation behaviour

## Abstract

**Background:**

Insecticide treated bed nets (ITN) are considered a core malaria vector control tool by the WHO and are the main contributor to the large decline in malaria burden in sub-Saharan Africa over the past 20 years, but they are less effective if they are not broadly and regularly used. ITN use may depend on factors including temperature, relative humidity, mosquito density, seasonality, as well as ideational or psychosocial factors including perceptions of nets and perceptions of net use behaviours.

**Methods:**

A cross–sectional household survey was conducted as part of a planned randomized controlled trial in Magoe District, Mozambique. Interviewers captured data on general malaria and ITN perceptions including ideational factors related to perceived ITN response efficacy, self-efficacy to use an ITN, and community norms around ITN using a standardized questionnaire. Only households with sufficient ITNs present for all children to sleep under (at least one ITN for every two children under the age of five years) were eligible for inclusion in the study. Additional questions were added about seasonality and frequency of ITN use.

**Results:**

One-thousand six hundred sixteen mother–child dyads were interviewed. Responses indicated gaps in use of existing nets and net use was largely independent of ideational factors related to ITNs. Self-reported ITN use varied little by season nor meaningfully when different methods were used to solicit responses on net use behaviour. Mothers’ perceived response efficacy of ITNS was negatively associated with net use (high perceived response efficacy reduced the log-odds of net use by 0.27 (95% CI − 0.04 to − 0.51), implying that stronger beliefs in the effectiveness of ITNs might result in reduced net use among their children.

**Conclusions:**

In this context, ITN use among children was not clearly related to mothers’ ideational factors measured in the study. Scales used in solicitation of ideation around ITN use and beliefs need careful design and testing across a broader range of populations in order to identify ideational factors related to ITN use among those with access.

## Background

Insecticide treated bed-nets (ITN) are considered a core vector control tool by the World Health Organization (WHO) and are believed to be the main contributor to the large decline in malaria burden in sub-Saharan Africa over the past 20 years [1, 2]. However, ITNs are less effective if they are not broadly and regularly used [3]. Net use is driven by a number of factors; most importantly, access to ITN within the household has clearly been shown to be the primary determinant of use in Sub-Saharan Africa [4]. Even when access to nets is sufficient, use may still be influenced by factors including temperature, relative humidity, mosquito and other nuisance insect density, as well as ideational or psychosicial factors including perceptions of nets and perceptions of net use behaviours. Ideational factors may include perceptions of the effectiveness of ITNs (response efficacy) and confidence in one’s ability to use an ITN (self-efficacy), and community norms around ITN use [5]. Other factors such as ITN shape, color and stitching patterns (which can relate to comfort) have not been shown to influence ITN use behaviour [6].

Ideation refers to a predictive model of behaviour change that examines how people think about and perceive behaviours. Ideational factors fall into three categories: cognitive, including beliefs, norms and self-efficacy; emotional, including fear and trust; and social, including support and influence. The more ideational factors that apply to an individual the more likely that individual is to engage in the target behaviour according to this model [7, 8]. One study using data from three cross-sectional surveys in Madagascar, Mali and Nigeria found meaningful though small associations between ideational factors and ITN use [7]. Ideational variables have also been shown to be influenced through the use of malaria communication campaigns [9]. However, the relationships between malaria ideation and net use are not consistent across locations, indicating that specific communication campaigns may need to be tailored to local contexts in order to be most effective [7, 9, 10]. Understanding the strength of association between the different ideational factors and the intended behaviour (e.g. net use) may be useful for effectively designing these tailored communication campaigns for maximum behavioural impact.

Storey et al. developed a series of scales covering various ideational factors related to malaria prevention, which was tested in a number of sub-Saharan African countries [7]. These scales were designed to measure malaria prevention related ideational factors consistent with a meta-theory of health communication first presented by Kincaid et al. [8]. This study examines the relationship between maternal responses on these scales and children’s bednet use in mother–child dyads from a cross-sectional survey deployed as the baseline for a community randomized controlled trial of interpersonal communication to improve bednet use in central Mozambique.

## Methods

### Study site

**Fig. 1:**
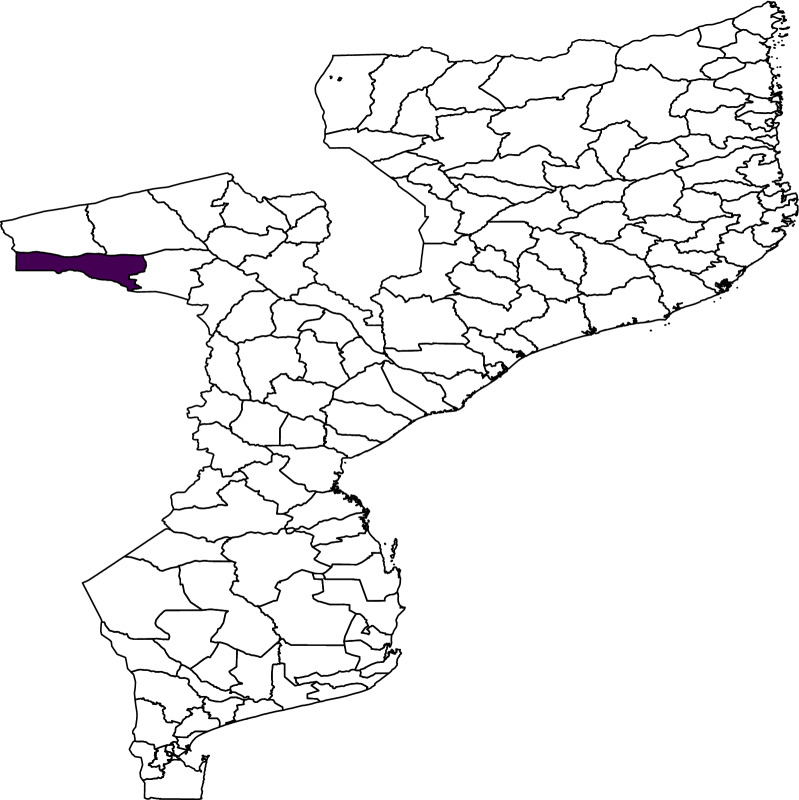
Magoe District, Mozambique

The study was conducted in Magoe District, Mozambique, an area in the north west of the country bordering both Zambia and Zimbabwe as well as the Cahora Bassa Lake and the Zambeze River (Fig. 1). The district is largely agricultural and sparsely populated with a population of less than 100,000 persons. The district was chosen as the proposed site of a community randomized controlled trial of interpersonal communication methods to improve the level and consistency of bed-net use because it was shown to have lower levels of net use than other parts of the country in previous cross-sectional surveys and was perceived to be an area in need of additional support for ITN use [11]. A convenience sample of one hundred twenty census enumeration areas were selected for use in the study. The selected areas are shown in Fig. 1.

### Enumeration and sampling

Prior to selection of households for participation in the household survey, full enumeration including geolocation of all households in the selected study enumeration areas was conducted. Household size and ITN ownership was determined during the enumeration. In each study enumeration area, 15 households meeting study inclusion criteria (having at least one child under 5 years of age, and at least one ITN for every two children under five years of age) were selected for participation in the household survey using simple random sampling stratified by study cluster.

### Survey design and content

The household survey followed a standard Malaria Indicator Survey design with addition of modules derived from the Malaria Behavior Survey, including a questionnaire for the household head or appropriate adult respondent which included general questions about the household, a household roster and an ITN roster [12]. A women’s questionnaire was also deployed to ask adult women of reproductive age additional specific questions including questions about children in their care. In addition to the standard elements of a Malaria Indicator Survey (MIS), additional modules included questions about the consistency of net use across season and about net use over the previous week as opposed to the night before the survey. In the women’s questionnaire a series of scales previously deployed by Storey et al. were used to assess women’s general attitudes toward malaria and ITNs, their perceived response efficacy of ITNs, perceived self-efficacy to properly utilize ITNs and perceptions of community norms regarding ITNs [7].

### Data analysis

Data was collected using a bespoke CSPro data entry form by trained surveyors. Data was cleaned using STATA (Version 16.0) and R. Analysis was conducted using R (Version 4.0.3). Univariate analysis and means and proportions are reported after accounting for survey clustering using the ’survey’ package [13]. Ideational scales were synthesized into uni-dimensional quantitative scales using polychoric principal components analysis using the ’psych’ package [14]. ‘’Don’t know” responses in scales were considered the intermediate level in likert style questions. Results from univariate scale analysis were presented using the ’likert’ package [15]. Ideational scales were dichotomized for multivariable analysis and summary reporting. Multivariable analysis was conducted using multi level logistic regression including random effects for study cluster as implemented using the ’lme4’ package [16]. In multi-variable analysis the reported use of ITN the night before the survey by children under 5 years of age was the outcome and characteristics of the household, mother, child and mother’s ideational responses were considered as potential predictors. We conducted a complete case analysis, meaning that observations with missing data in outcome or any predictor were not included in the multivarible model. Model selection was performed based on theoretical considerations and Akikae’s Information Criterion. Scale cohesion was assessed using Cronbach’s alpha calculated using the ’psych’ package [14].

## Results

Women’s responses on the women’s questionnaires were linked to ITN use among their children, leaving a final sample of 1616 mother child dyads (Table 1). Net use among children in the matched dyads was 50.1% (95% C.I. 46.4–54.0%). Net use on the night before the survey was more common among children whose families had higher socioeconomic status and whose mothers had been exposed to malaria behavioural change communication prior to the study (Table 2).

**Table 1:** Basic sample characteristics

	N (%)
N Total	1616
High perceived norms (%)	818 (56.2)
High response efficacy (%)	804 (55.4)
High perceived self efficacy (%)	979 (60.6)
High ITN attitudes (%)	833 (51.5)
High general ideation (%)	738 (45.7)
Received malaria BCC (%)	296 (18.3)
Wealth quintile (%)	
Poorest	410 (25.4)
2nd Poorest	305 (18.9)
Middle	340 (21.0)
2nd Wealthiest	292 (18.1)
Wealthiest	269 (16.6)
Child slept under net last night (%)	809 (50.1)

**Table 2:** Determinants of child net use (logistic regression)

	Coef. (SE)
2nd Lowest wealth quintile	0.014
	(0.184)
Middle wealth quintile	− 0.052
	(0.179)
2nd Highest wealth quintile	0.227
	(0.191)
Highest weath quintile	0.416$^{*}$
	(0.218)
High general ideation	0.222$^{*}$
	(0.124)
High perceived self efficacy	0.217$^{*}$
	(0.131)
High response efficacy	− 0.269$$^{**}$$
	(0.133)
High ITN attitudes	− 0.117
	(0.130)
High percieved community norms	0.034
	(0.129)
Prior expossure to malaria BCC	0.138
	(0.162)
Constant	− 0.208
	(0.200)
Observations	1303
Log likelihood	− 877.252
Akaike Inf. Crit.	1778.505
Bayesian Inf. Crit.	1840.574

*Notes:* NA

$$^{**}$$Significant at the 5 percent level.

$^{*}$Significant at the 10 percent level

**Fig. 2:**
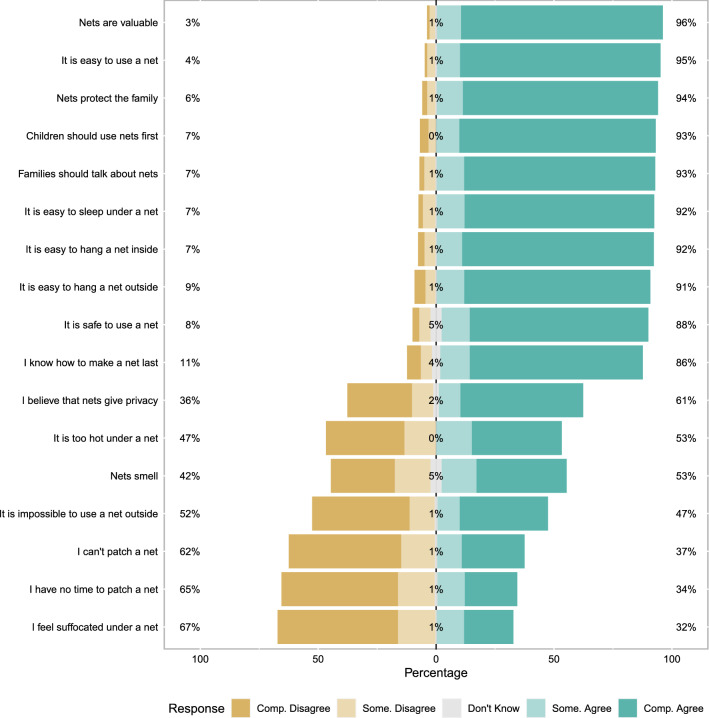
Responses to scale questions regarding general attitudes towards insecticide treated bed-nets. Numeric labels show percent positive (agree, somewhat agree), percent neutral and percent negative (disagree, somewhat disagree) responses

**Fig. 3:**
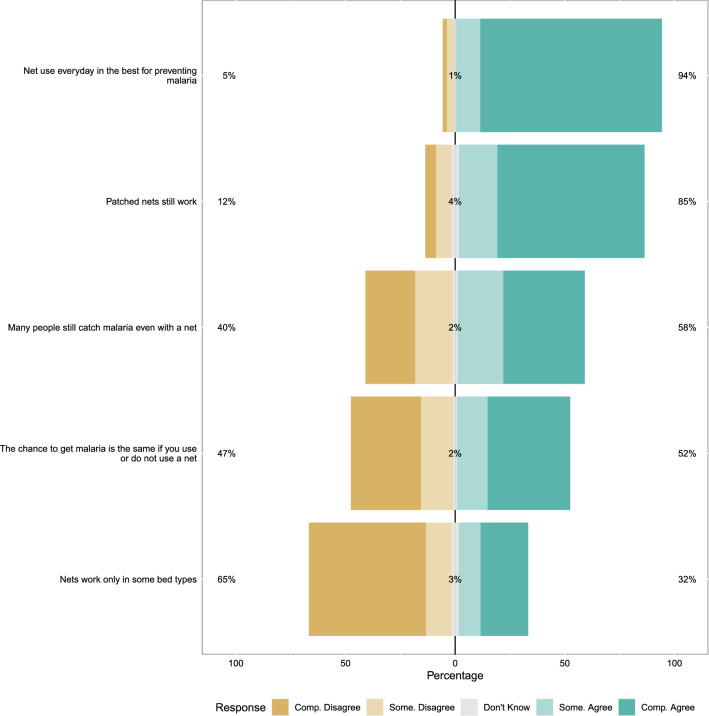
Responses to scale questions regarding ITN response efficacy. Numeric labels show percent positive (agree, somewhat agree), percent neutral and percent negative (disagree, somewhat disagree) responses

**Fig. 4:**
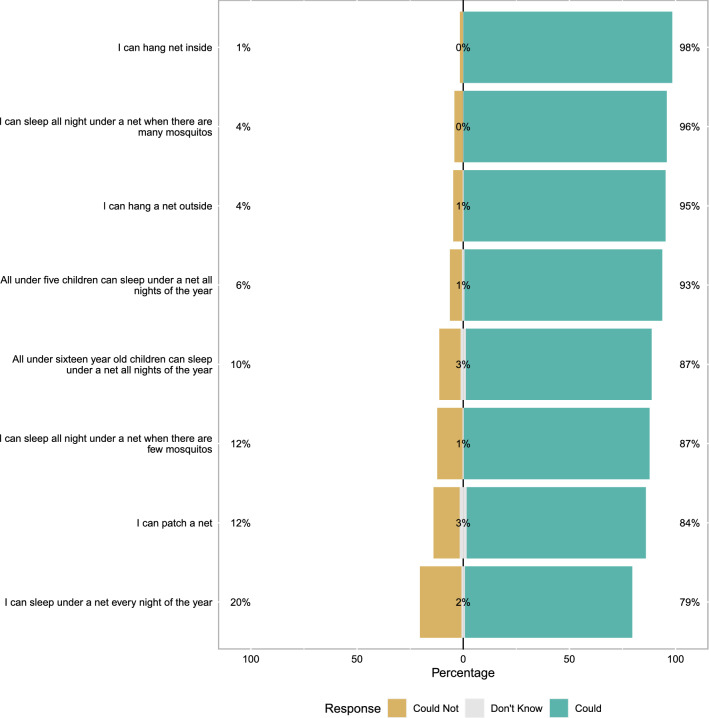
Responses to scale questions regarding ITN perceived self-efficacy

**Fig. 5:**
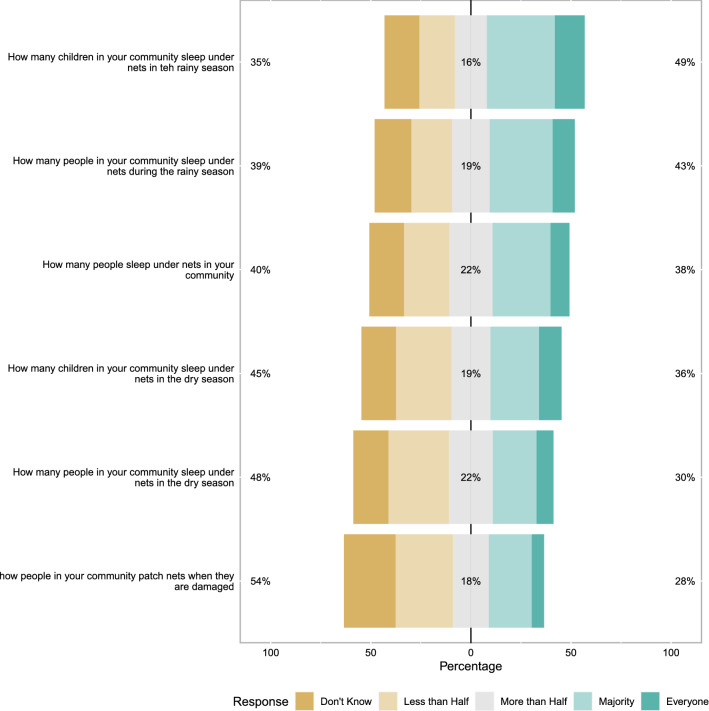
Responses to scale questions regarding ITN perceived norms. Numeric labels show percent positive (agree, Somewhat agree), percent neutral and percent negative (disagree, somewhat disagree) responses

General ITN and malaria knowledge was fairly high and negative perceptions of nets were present but not common (Fig. 2). The general malaria and ITN scale overall had relative modest Cronbach’s alpha (0.62), indicating only moderate cohesion. Similarly, overall perceptions of ITN response efficacy were also high and negative perceptions of response efficacy were generally minority views. For example, only 32% of mothers agree (somewhat or completely) that nets only work for some bed types. Again the Cronbach’s alpha for this scale was only modest (0.73) (Fig. 3). ITN perceived self-efficacy was high, 80% of mothers reported that they could sleep under a net every night of the year (Cronbach’s alpha 0.67) (Fig. 4). Perceptions of community norms were more varied than other responses with similar numbers of respondents indicating that majorities of the members of their communities engaged (or failed to engage) in specific ITN related behaviours (Fig. 5). This scale also showed the highest degree of coherence with Cronbach’s alpha of 0.92. A final sample of 1303 mother-child dyads with complete ideational responses were considered for multi-variable analysis. The final model is shown in Table 2. Overall, higher household socioeconomic status was associated with significantly higher net use, while higher maternal ITN response efficacy was associated with less ITN use by their children. Other ideational factors did not show any significant association with children’s net use. Respondents reported that they were slightly more likely to use nets during the wet season than the dry season (OR 1.26 95% C.I. 1.12–1.42 $\textit{p} < 0.001$) though the absolute difference was small (61.6% in the wet season and 55.9% in the dry season). Responses to questions about net use during the previous week were largely consistent with reported net use on the night before the survey. A mixed effects logistic model using the previous week reported net use was a highly effective classifier of net use on the previous night (AUC = 0.906 95% C.I. 0.894–0.918).

## Discussion

A cross-sectional household survey in Magoe district, Mozambique was used to assess general malaria and ITN perceptions, including perceived ITN response efficacy, self-efficacy to use ITNs, and community norms of net use. Use of ITN among children with access to ITN at the household level in this context is relatively modest with only half of children reporting to have used a net the previous night. This level of net use among children is much lower than the 73% net use that was found in the 2018 MIS. This discrepancy in net use is mostly likely due to the timing of the two surveys; the Magoe survey was conducted at the end of the dry season (in November 2019) when mosquito densities are typically at their lowest, while the 2018 MIS was conducted at the end of the rainy season when mosquito densities, as well as perceived risk of malaria, are typically at their highest. The difference may also be related to the differing sample and timing of the surveys. Socioeconomic status was shown to be a strong predictor of ITN use when households had access to ITN in this study. This finding highlights on going inequity in malaria prevention, despite high and equitable access among the study population. Unexpectedly, higher maternal response efficacy of nets was associated with lower use of nets by children during the dry season. While this result is somewhat surprising, similar phenomena have been shown in other domains, such as student performance, whereby stronger perceptions of self-efficacy or overconfidence is negatively associated with desired outcomes [17]. This phenomenon might arise in this case because the individuals sense that they can achieve their desired outcomes without rigorous use of the intervention as the efficacy is perceived to be high. Storey and colleagues found this precise result in cross sectional data from Nigeria [7]. Overall, other ideational responses were not strongly associated with ITN use among this group where ITN access was universal. This finding suggests that messaging to reinforce the importance of consistent net use (*i.e.* sleeping under a net every night) in order to achieve the highest level of protection from nets could improve net use in households with a sufficient number of nets, especially during the dry season when mosquito numbers are much lower.

It is well–documented that access to an ITN is one of the most, if not the single most, important determinants of net use [4, 7, 10]. The inclusion criteria for this study ensured that population access to nets was near universal in the study population. While other studies have identified various ideational factors about net use as predictive of use, they generally looked at populations in which net access was not universal [7]. It is possible that ideational factors may work further upstream, mainly by influencing families and households to ensure that they have access to ITNs and thus their impact on net use is largely mediated by net access [9]. If this is the case, then it is not tremendously surprising that in a population where access to ITNs is universal that ideational factors would be less associated with variability in ITN use.

In this study population, the coherence of the ITN ideational scales were only moderate, with Cronbach’s alpha scores ranging from 0.5 to 0.9. The scales, which have also been used in other settings, might need refinement or adjustment for use in different study populations [7]. The scales employed here also include a ‘’Don’t Know” response. Future applications of these or related scales should not include a ‘’Don’t Know” category and rather encourage respondents to respond with the category that most fits their individual perceptions as such responses do not fit easily into the Likert–type responses used in these scales.

Use of nets by children is not necessarily the same as adoption of health behaviours overall, including net use by adults. Ideational models of behaviour change with measurement of ideational factors among mothers may translate imperfectly to choices about net use for their children. Additional constraints to decision–making may be present at the household level, such as a father’s influence or additional older children needing bednets as well. The translation of measurements about a mother’s individual beliefs to health decision making for her children may introduce additional opportunities for ideation and action to diverge. Clearly, a mother’s beliefs and intentions are expected to be highly influential as to child net usage, but it remains to be seen the extent to which these particular measures of ideation can effectively capture this relationship in this setting. Future use of similar scales and analyses should consider household decision-making autonomy and risk-perception as additional ideational factors that may be context-specific. This study did not find significant statistical associations between net use and key ideational factors, but did in fact find an unexpected signifcant negative association between mother’s ITN response efficacy and child net use. The fact that access to ITNs in this population is universal but there were still substantial gaps in ITN use by children under five years old points to as-yet unidentified behavioural factors preventing net use in this population. Future data collection to understand these behavioural barriers could incorporate mixed-methods approaches to complement this study based on the same ideational framework. The population selected for inclusion in this trial was not a representative sample of Magoe District as a whole and as such the results here should not be considered as estimates of district level net use, or of other population level parameters. The associations between net use and other factors however are likely to extend to all households with sufficient access to ITNs within Magoe District. These findings may be helpful in the design of behaviour change communication strategies designed to influence mother-child ITN use patterns in similar settings.

## Conclusions

Net use is modest at only 50% among sampled households in Magoe District, Mozambique, during the dry season, when malaria risk is at its lowest. However, these results suggest that net use among children was not greatly influenced by the perceptions of mothers in net owning households in this setting. High ITN response efficacy may be inversely related to ITN use among children in households with sufficient ITN access, suggesting that reinforcement of consistent use to ensure efficacy might be an important component of future social and behaviour change campaigns. Ideational and perceptional questionnaires may be useful for identifying and measuring perceptions about net use and beliefs but need careful consideration and refinement before scales are considered standard and are widely applied.

## Data Availability

The data that support the findings of this study are available from the corresponding author, JY, upon reasonable request.
